# Torque measurements reveal large process differences between materials during high solid enzymatic hydrolysis of pretreated lignocellulose

**DOI:** 10.1186/1754-6834-5-57

**Published:** 2012-08-06

**Authors:** Benny Palmqvist, Gunnar Lidén

**Affiliations:** 1 Department of Chemical Engineering, Lund University, Box 124, Lund, SE-221 00, Sweden

**Keywords:** Lignocellulose hydrolysis, Mixing, Torque, Power input

## Abstract

**Background:**

A common trend in the research on 2^nd^ generation bioethanol is the focus on intensifying the process and increasing the concentration of water insoluble solids (WIS) throughout the process. However, increasing the WIS content is not without problems. For example, the viscosity of pretreated lignocellulosic materials is known to increase drastically with increasing WIS content. Further, at elevated viscosities, problems arise related to poor mixing of the material, such as poor distribution of the enzymes and/or difficulties with temperature and pH control, which results in possible yield reduction. Achieving good mixing is unfortunately not without cost, since the power requirements needed to operate the impeller at high viscosities can be substantial. This highly important scale-up problem can easily be overlooked.

**Results:**

In this work, we monitor the impeller torque (and hence power input) in a stirred tank reactor throughout high solid enzymatic hydrolysis (< 20% WIS) of steam-pretreated *Arundo donax* and spruce. Two different process modes were evaluated, where either the impeller speed or the impeller power input was kept constant. Results from hydrolysis experiments at a fixed impeller speed of 10 rpm show that a very rapid decrease in impeller torque is experienced during hydrolysis of pretreated arundo (i.e. it loses its fiber network strength), whereas the fiber strength is retained for a longer time within the spruce material. This translates into a relatively low, rather WIS independent, energy input for arundo whereas the stirring power demand for spruce is substantially larger and quite WIS dependent. By operating the impeller at a constant power input (instead of a constant impeller speed) it is shown that power input greatly affects the glucose yield of pretreated spruce whereas the hydrolysis of arundo seems unaffected.

**Conclusions:**

The results clearly highlight the large differences between the arundo and spruce materials, both in terms of needed energy input, and glucose yields. The impact of power input on glucose yield is furthermore shown to vary significantly between the materials, with spruce being very affected while arundo is not. These findings emphasize the need for substrate specific process solutions, where a short pre-hydrolysis (or viscosity reduction) might be favorable for arundo whereas fed-batch might be a better solution for spruce.

## Background

Lignocellulosic biomass is widely regarded as a promising feedstock for the production of biofuels such as bioethanol, which has the potential to reduce oil dependency and thus also greenhouse gas emissions [1]. The technology required to convert lignocellulosic biomass into sugars and subsequently ferment them into ethanol is considered relatively mature, as evidenced by the construction of numerous demonstration and full scale plants throughout the world [1,2]. In order to make lignocellulosic ethanol cost-competitive, however, a high amount of water insoluble solids (WIS) is needed in the process. This reduces the capital investment costs and results in higher ethanol titers, which in turn, lowers the energy demand in the following distillation step, resulting in significant economic benefits [3].

Nonetheless, increasing the WIS content is not without problems and recent studies, summarized by Kristensen *et al.*[4], indicate a negative correlation between glucose yield and increased solids loading. These studies cover different types of raw material (e.g. wheat straw, corn stover, olive tree, softwood) and mixing systems (e.g. rotating drums and stirred tanks) and give the impression that the influence of an increasing WIS content vary depending on these factors. Maintaining a well-mixed process (e.g. to adequately control pH and temperature) is more difficult at increased WIS content, since the viscosity of the thick fiber-slurries increases rapidly when the concentration increases [5-7]. Furthermore, at elevated viscosities, the power input needed to sufficiently mix the slurry might increase to unacceptably high levels. Zhang *et al.*, for example, reports mixing energy inputs equal to as much as 59% of the higher heating value of the produced ethanol at 30% dry matter [8], corresponding to a mean power input of 3.9 kW/ton slurry. In their study a helical ribbon impeller was employed at a relatively high impeller speed of 120 rpm. Dasari *et al.* were however able to achieve adequate mixing, at only 2 rpm, by utilizing a horizontal scraped surface bioreactor and hence the required power input was greatly reduced to a maximum of 0.56 kW/m^3^ slurry at 25% solids loading [9].

Recent studies on the rheology of pretreated biomass slurries have mainly focused on corn stover slurries [6,10], although other biomasses have been investigated, including red-oak [11] and spruce [5]. Biomass slurries are, in general, shear thinning and highly viscous, although the problems related to high viscosities will be most pronounced during the start of the hydrolysis as the enzymatic degradation of the biomass reduces the viscosity over time. Measuring the viscosity of pretreated biomass at relevant concentrations is not trivial, mainly due to the existence of solid fibers in the materials. In common rheometers particle settling and particles getting stuck in the measuring well are problems encountered. A more practical approach to estimate relative viscosities could therefore be to measure the impeller torque during mixing of the material. It is known that, under laminar flow conditions, the viscosity of the liquid is directly proportional to the impeller torque, according to equation 1, presented by Pimenova *et al.*[10],

(1) µ
app=2πMcNiDi3

where μ_app_ is the apparent viscosity of the liquid, D_i_ the impeller diameter, N_i_ the impeller speed, c an empirically determined constant and M is the torque. Thus by measuring the torque during enzymatic hydrolysis, the viscosity changes can be followed. Although the rheology of pretreated biomass is better and better understood, research on the specific influence of rheology on enzymatic hydrolysis remains surprisingly few.

We have previously conducted an extensive rheological characterization of pretreated spruce material [5] and coupled this to the effects of mixing on the enzymatic hydrolysis [12]. Our study showed that impeller speed (i.e. stirring power input) affects the hydrolysis rate and yield of that particular pretreated spruce to a surprisingly large extent (about 100% yield increase going from 75 to 500 rpm) [12]. These findings were supported by reports from studies on Avicel [13] and spruce [14] in stirred tanks, whereas contradictory results have been reported for other materials. For example, in their studies on high solid enzymatic hydrolysis in rotating drums, Roche *et al.*[15] and Jorgensen *et al.*[16] argue that once the enzymes are evenly distributed, the influence of mixing is negligible. However, these studies were conducted with agricultural residues, i.e. corn stover and wheat straw respectively, as opposed to softwood (e.g. spruce). In general it is therefore difficult to compare results of mixing studies with different materials, since often also different equipment has been used and this will likely affect the results.

In the current study, the influence of mixing on the enzymatic hydrolysis of different types of pretreated materials, i.e. the energy crop *Arundo donax* (giant reed) and the softwood *Picea abies* (spruce), was studied using a stirred tank reactor. Torque and power consumption was monitored throughout the enzymatic hydrolysis at different WIS contents, which allows the changes in rheology during the hydrolysis to be followed. A key finding is that during hydrolysis, spruce retains its fiber network strength (i.e. viscosity) much longer than arundo, resulting in a substantially larger mixing power input for pretreated spruce. This difference between the two materials primarily becomes evident as the WIS content is increased.

## Materials and methods

### Raw material and pretreatment

Two different materials were used in the study. Steam pretreated *Arundo donax* was kindly provided by Chemtex (Tortona, Italy). The material was pretreated at the Chemtex pilot facilities and shipped (and stored) frozen until used. Wood chips (2–10 mm) from debarked Norway spruce (*Picea Abies*) were provided by Witskövfle Sågverk AB, Sweden. The wood chips were impregnated with 2.5% SO_2_ (based on moisture content) for 15 min in plastic bags. The material was then steam pretreated for 5 min at 210°C in a 10 L reactor, as previously described by Palmqvist *et al.*[17]. The pretreated spruce was stored at 4°C until used. The composition of the pretreated materials (Table 1) was analyzed using NREL (National Renewable Energy Laboratories) standard procedures [18] and the WIS content of each material was determined by washing the fibers repeatedly with deionised water over filter paper (Whatman No. 1). The WIS contents of the pretreated materials were measured to 36.5% (w/w) and 12.7% (w/w) for arundo and spruce respectively.

**Table 1:** Fiber composition of pretreated arundo and spruce

**Arundo (% of WIS)**		**Spruce (% of WIS)**	
Glucan	50.2%	Glucan	42.9%
Mannan	n.d.*	Mannan	n.d.
Galactan	n.d.	Galactan	n.d.
Xylan	6.4%	Xylan	n.d.
Arabinan	n.d.	Arabinan	n.d.
Lignin	36.8%	Lignin	46.0%

The WIS content was measured to 36.5% and 12.7% respectively. (* n.d. = not detected, i.e. below the sensitivity of the instrument).

### Hydrolysis experiments

All hydrolysis experiments were carried out in duplicates in a 3 L bioreactor (Belach Bioteknik, Stockholm, Sweden) with an inner diameter of 14 cm, at a working weight of 1.5 kg. The reactor was equipped with an anchor impeller with a diameter (D_i_) of 13 cm and a blade width (w_i_) of 2 cm. The reactor was equipped with a strong motor (down-geared fivefold) able of measuring the torque on the stirrer shaft which allowed monitoring of torque and power input throughout the hydrolysis. Experiments were performed at 10, 15 and 20% WIS content for both arundo and spruce and the desired WIS content was reached by diluting the pretreated material with DI-water. Since the WIS content of the pretreated spruce was 12.7%, liquid was separated in a filter press until a WIS content of about 28% was reached. The fiber cake was then resuspended in the original slurry, in order to reach starting WIS concentrations of 15 and 20% WIS respectively. The pH of the slurries was manually set to 5.0 by the addition of 12 M NaOH before starting the experiments, and then left uncontrolled. Sodium azide (0.4 g L^-1^) was added to the arundo slurries before starting the hydrolysis in order to prevent microbial contamination. The spruce material was rather toxic itself and hence required no sodium azide addition. The enzyme preparation used was Cellic CTec2 (kindly provided by Novozymes A/S – Bagsvaerd, Denmark) at a loading of 0.1 g solution g^-1^ WIS. Samples for HPLC-analysis were taken repeatedly throughout the hydrolysis process.

### Analysis

HPLC was used for the sugar analysis. Samples from the hydrolysis liquid were centrifuged in 2 mL Eppendorf tubes at 13,000 rpm for 5 min. (Z 160 M, HERMLE Labortechnik, Wehingen, Germany). The supernatant was then filtered through 0.2 μm filters, and stored at −20°C until analyzed. The sugars concentrations, mainly glucose, were determined using a polymer column (Aminex HPX-87P, Bio-Rad Laboratories, München, Germany) at an operating temperature of 85°C. Deionized water (Elga Maxima, Elga, Marlow, UK) was used as eluent, with a flow rate of 0.6 mL min^-1^. The sugars were detected with a refractive index detector (Waters 2410, Waters, Milford, MA, USA).

### Yield calculations

Glucose yields are often calculated according to equation 2 where only the measured sugar concentrations are accounted for. This approximation is rather accurate for hydrolysis of diluted fiber suspensions (< 5–10% WIS) but for more concentrated suspensions this estimation has been shown to significantly overestimate the yields [6,19,20]. When considering the glucose yields a number of different factors need to be accounted for, e.g. that the amount of liquid decreases during hydrolysis due to the incorporation of water into the sugar molecules and that the density of the liquid increases proportionally to the sugar concentrations. These factors become significant at high solids content and therefore a more elaborate method is needed to accurately calculate the yield based on measured concentrations. In this work, the glucose yields were calculated according to equation 3, proposed and verified by Zhu *et al.*[20], which takes into account both the decrease in liquid amount and the increase in liquid density throughout the hydrolysis. It should be noted that densities were estimated according to the model proposed by Zhu *et al.*[20] and not measured. The impact of this is, however, considered rather low since it was shown to have a larger effect if washed fibers were used instead of the whole slurry. The initial oligomer concentrations were measured using standard NREL procedures [21].

(2) $$Y_{g}=\frac{C_{g}-C_{g0}}{\phi_{g}C_{is0}X_{G0}}\;$$

(3) $$Y_{g}=\frac{\left[C_{g}\left(\frac{V_{h}}{V_{h0}}\right)-C_{g0}\right]\left(\frac{1}{f_{ts0}X_{is0}}-1\right)}{\phi_{g}\rho_{h0}X_{G0}+\phi_{\mathrm{gos}}C_{gos0}\left(\frac{1}{f_{ts0}X_{is0}}-1\right)}\;$$

The nomenclature for the equations is presented in Table 2.

**Table 2:** **Nomenclature for parameters in equations 2 and 3**

**Y**_**g**_	**Glucose yield (of theoretical maximum)**	**f**_**tso**_	**Initial mass fraction of total solids (soluble + insoluble) in slurry**
C_g_	Glucose concentration (g/L)	ρ_h0_	Initial density of liquid (g/L)
C_g0_	Initial glucose concentration (g/L)	V_h_	Volume of hydrolysate liquid (L)
C_gos0_	Initial concentrations of glucose oligomers (g/L)	V_h0_	Initial volume of hydrolysate liquid (L)
X_g0_	Initial mass fraction of glucan in insoluble solids	ϕ_g_	Molecular ration of glucose to glucan; ϕg = 180/162 = 1.11
X_is0_	Initial mass fraction of insoluble solids in total solids	ϕ_gos_	Ratio of glucose molecular weight to average monomer weight of glucose oligomers; ϕgos = 180/166.5 = 1.08 (assuming glucose oligomers have an average DP of 4)

## Result and discussion

To evaluate the differences between the fast growing energy crop, arundo, and the common softwood spruce, hydrolysis experiments were conducted at three different WIS loadings (10, 15 and 20%). Furthermore, two different process options were assessed, where either the impeller speed or the impeller power input was keep constant. This was done since a large discrepancy was found between the two materials when operating at a fixed impeller speed, both with respect to glucose yields and torque/power inputs during the process. A substantially larger total energy input for operating the impeller at fixed speed was found for spruce compared to arundo (at similar WIS content).

### Effects of an increase in WIS content at constant impeller speed

**Figure 1:**
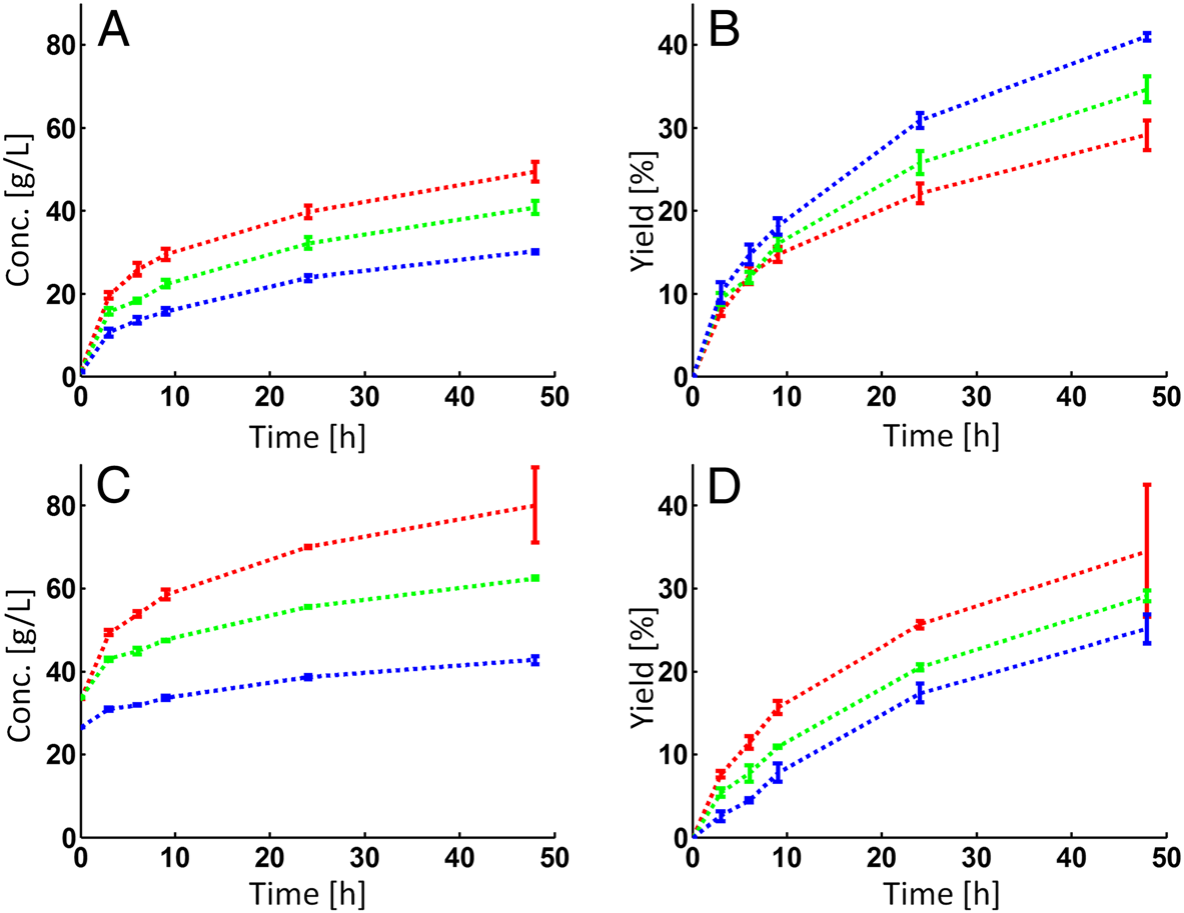
**Enzymatic hydrolysis of arundo and spruce at constant impeller speed.** Glucose concentrations (**A**,**C**) and yields (**B**,**D**) during enzymatic hydrolysis of arundo (**A**,**B**) and spruce (**C**,**D**). Impeller speed was kept constant at 10 rpm throughout the hydrolysis. Blue = 10% WIS, Green = 15% WIS and Red = 20% WIS.

During enzymatic hydrolysis of arundo, it was found that increasing the WIS content from 10 to 20% (keeping a constant impeller speed of 10 rpm) resulted in increased concentrations of sugars in the hydrolysate liquid (Figure 1A). However, as expected, the glucose yields decreased at higher WIS contents as seen in Figure 1B and Table 3. Performing the same experiments with pretreated spruce revealed that an increase in WIS content resulted not only in increased sugar concentrations (Figure 1C), but, unexpectedly, also in improved glucose yields (Figure 1D and Table 3).

**Table 3:** Summary of all hydrolysis experiments after 48 hour of hydrolysis. The total energy input was calculated by integrating the measured power input over the 48 hour hydrolysis. Averaged power inputs where then calculated from the total energy inputs

	**Arundo controlled speed (10 rpm)**				**Spruce controlled speed (10 rpm)**			
	**Glucose yield (% of maximum)**	**Average power input (W)**	**Total energy input (kJ)**	**Total energy input (kJ/kg initial WIS)**	**Glucose yield (% of maximum)**	**Average power input (W)**	**Total energy input (kJ)**	**Total energy input (kJ/kg initial WIS)**
**10**% **WIS**	41.0 ± 0.5	0.081 ± 0.010	14.1 ± 1.7	102 ± 11.2	25.1 ± 1.7	0.10 ± 0.004	16.8 ± 0.7	115.7 ± 5.2
**15**% **WIS**	34.6 ± 1.5	0.090 ± 0.008	15.6 ± 1.4	64.8 ± 6.3	29.0 ± 0.6	0.18 ± 0.005	31.1 ± 0.9	135 ± 4.0
**20**% **WIS**	29.1 ± 1.8	0.106 ± 0.009	18.4 ± 0.2	60.8 ± 0.5	34.4 ± 7.9	0.51 ± 0.05	87.4 ± 7.9	272 ± 26.5
	**Arundo controlled power input**				**Spruce controlled power input**			
	**Glucose yield (% of maximum)**	**Controlled power input (W)**	**Total energy input (kJ)**	**Total energy input (kJ/kg initial WIS)**	**Glucose yield (% of maximum)**	**Controlled power input (W)**	**Total energy input (kJ)**	**Total energy input (kJ/kg initial WIS)**
**10**% **WIS**	40.8 ± 0.1	0.100 ± 0.002	17.3 ± 0.3	115 ± 2.0	44.0 ± 0.2	0.453 ± 0.004	78.3 ± 0.7	522 ± 4.7
**15**% **WIS**	35.1 ± 0.5	0.170 ± 0.003	29.3 ± 0.5	130 ± 2.2	34.4 ± 0.6	0.455 ± 0.002	78.6 ± 0.5	348 ± 2.0
**20**% **WIS**	30.3 ± 0.4	0.453 ± 0.001	78.3 ± 0.2	265 ± 0.7	27.6 ± 1.1	0.443 ± 0.001	76.6 ± 0.06	255 ± 0.2

**Figure 2:**
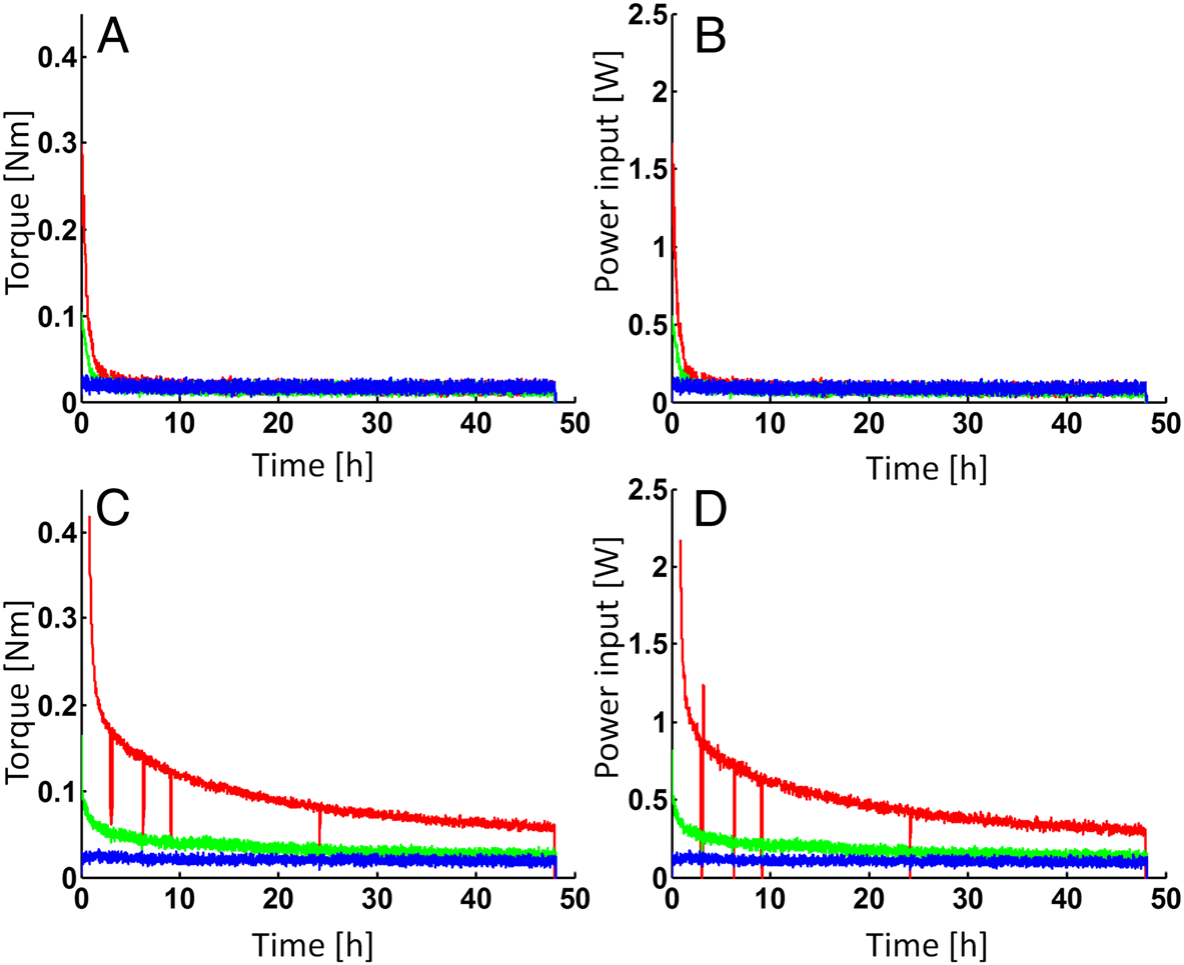
**Torque profiles and mixing power during enzymatic hydrolysis of arundo and spruce at constant impeller speed.** Torque (**A**,**C**) and power inputs (**B**,**D**) during enzymatic hydrolysis of arundo (**A**,**B**) and spruce (**C**,**D**). Impeller speed was kept constant at 10 rpm throughout the hydrolysis. Blue = 10% WIS, Green = 15% WIS and Red = 20% WIS.

The torque profiles during enzymatic hydrolysis at constant impeller speed differed significantly between arundo (Figure 2A) and spruce (Figure 2C). When hydrolyzing arundo, a rapid decrease in torque, due to fiber degradation, was observed and within the first 2–6 hours the slurries behaved similar regardless of their initial WIS content (Figure 2A). These results are in line with what Dasari *et al.* previously reported on the behavior of corn stover [9]. The quick drop in torque indicates that the viscosity changes rapidly during the first few hours of hydrolysis and then remains rather constant (according to equation 1). One should also note that the WIS content of the 20% initial arundo slurry never went below about 16–17% WIS (~30% conversion) which indicates that it is not merely the amount of insoluble solids that determines the rheology but also the structure/composition of the material. Since torque and hence power consumption decreased rapidly in the beginning, the total energy input over the whole 48 hours of hydrolysis were almost independent of the WIS concentration at the start of the process (Figure 2B and Table 3).

In contrast, the decrease in torque and power input during spruce hydrolysis was much slower with time, which consequently led to rather large discrepancies in total energy input between the different starting WIS contents (Figure 2C-D and Table 3). It should be noted that the pretreated spruce contains a higher amount of lignin compared to the arundo (Table 1). The lignin structure is not broken down during the hydrolysis and might therefore contribute more to retaining the higher viscosities of the spruce material.

**Figure 3:**
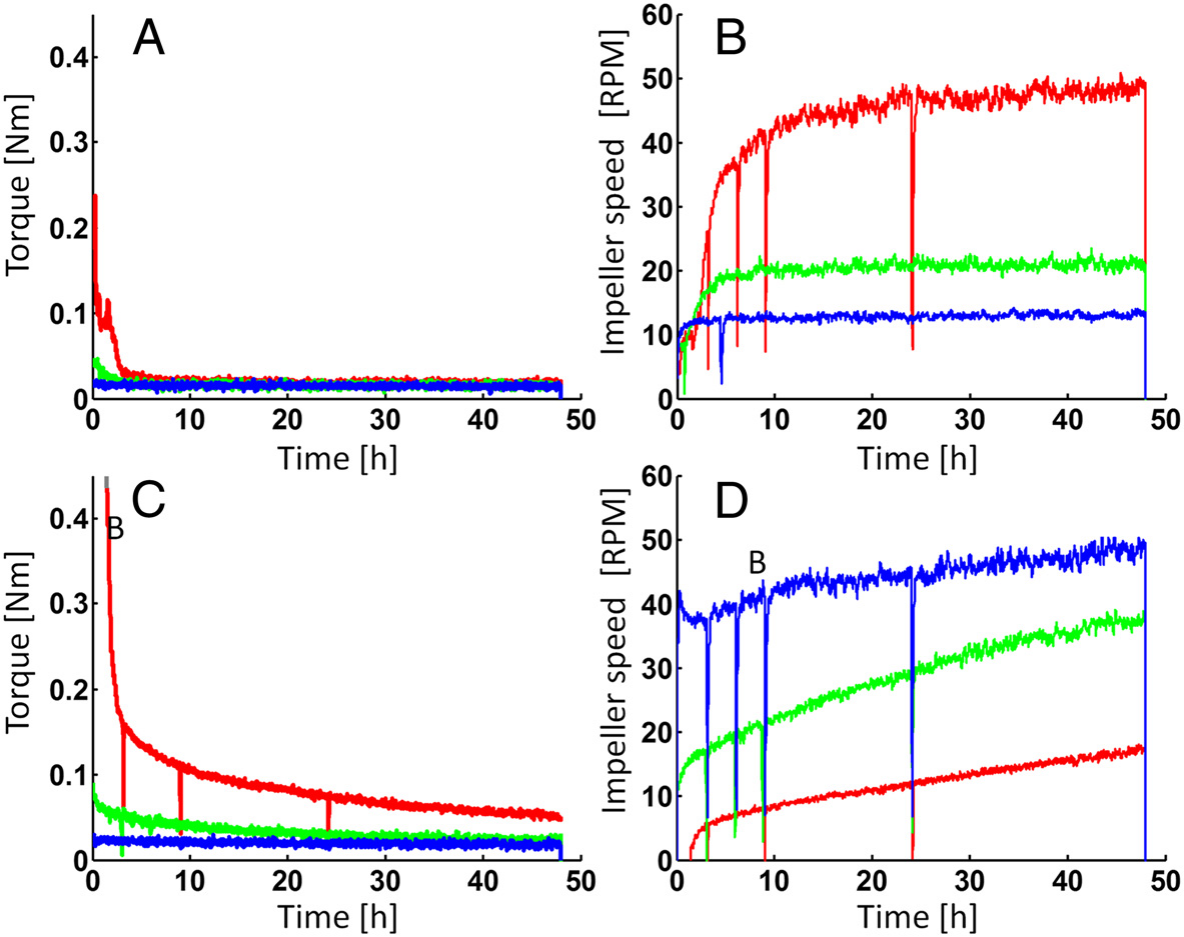
**Torque and impeller speed profiles during enzymatic hydrolysis of arundo and spruce at controlled power inputs.** Torque (**A**,**C**) and impeller speed (**B**,**D**) during enzymatic hydrolysis of arundo (**A**,**B**) and spruce (**C**,**D**) at controlled power input. For around (**A**,**B**), the power input was kept constant at 0.10 W for 10% WIS, 0.17 W for 15% WIS and 0.46 W for 20% WIS. All spruce experiments were performed at a constant power input of 0.46 W regardless of WIS content. Blue = 10% WIS, Green = 15% WIS and Red = 20% WIS.

A visual examination of pretreated arundo and spruce reveals that the fiber structure and the ability to retain water within the fibers differ across the materials. Arundo comprises rather large fibers and appears to be a “slurry of suspended solids” whereas spruce behaves more “paste-like” at similar WIS content. The rather fragile fiber network of arundo quickly loses most of its structure after the enzyme addition, as shown during the torque measurements (Figures 2A and 3A). The pretreated spruce on the other hand, consists of shorter fiber fragments and appears to retain its fiber network strength (i.e. viscosity) during a longer period of time, as seen by the high torque needed to mix the material (Figures 2C and 3C). Consequently, a higher power input (Figure 2D) and a much higher overall energy input (Table 3) is needed, compared to the hydrolysis of arundo.

### Effects of keeping a constant power input during hydrolysis

Since power input varied to a large extent between the two materials, a second set of experiments were conducted where the power input rather than the impeller speed was kept constant. The power inputs were controlled using a feed-back controller operating on the impeller speed throughout hydrolysis so that the impeller speed increased when the viscosity of the material (i.e. torque) decreased due to fiber degradation. In order to get a spruce hydrolysis comparison on an equal energy input basis, similar to the first set of arundo experiments, the hydrolysis experiments were carried out at the same controlled power input at different WIS contents, i.e. giving a higher total energy input/kg WIS at lower WIS contents. A constant power input of 0.46 W was chosen since it represented the calculated average power consumption for the first hydrolysis experiment with spruce at 20% WIS and 10 rpm. The arundo hydrolysis was instead performed a at fixed power input that increased with the starting WIS content in order to see if more power would increase the glucose yield, as it did for spruce. Power inputs of 0.10, 0.17 and 0.46 W (for 10, 15 and 20% WIS content respectively) were chosen since they represent the corresponding mean power inputs for the first set of spruce hydrolysis at 10 rpm (Figure 2D).

**Figure 4:**
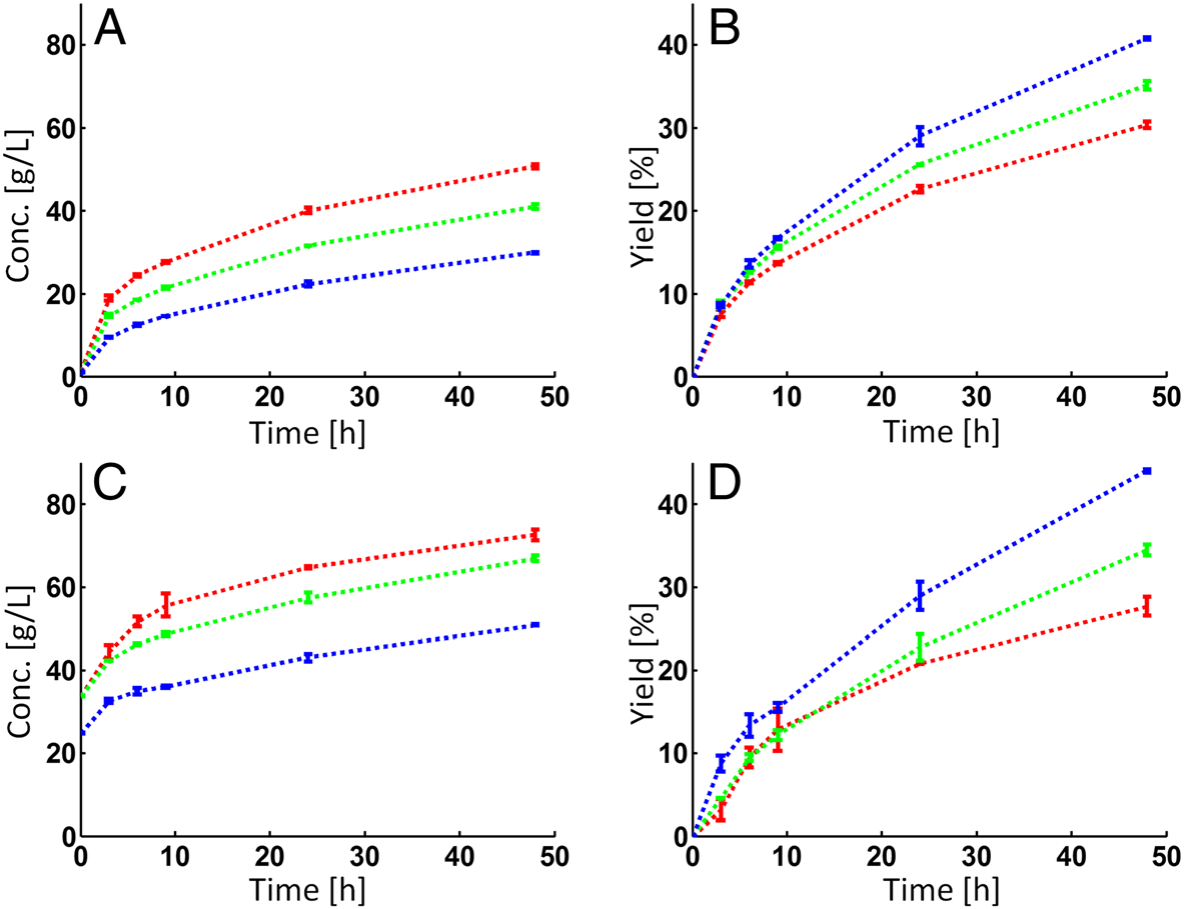
**Enzymatic hydrolysis of arundo and spruce at controlled power inputs.** Glucose concentrations (**A**,**C**) and yields (**B**,**D**) during enzymatic hydrolysis of arundo (**A**,**B**) and spruce (**C**,**D**). For around (**A**,**B**), the power input was kept constant at 0.10 W for 10% WIS, 0.17 W for 15% WIS and 0.46 W for 20% WIS. All spruce experiments were performed at a constant power input of 0.46 W regardless of WIS content. Blue = 10% WIS, Green = 15% WIS and Red = 20% WIS.

The glucose yields for arundo at fixed power inputs (Figure 4B) did not differ significantly from that at constant impeller speed (Figure 1B), as can be seen in Table 3. This despite the fact that the total energy input (both kJ and kJ/kg WIS) was higher at high WIS contents. Again, a very quick drop in torque (i.e. viscosity) is experienced during the initial stages of the hydrolysis (Figure 3A) as well as the rather WIS independent torque after a few hours. Since power input is directly proportional to both torque and impeller speed a corresponding increase in impeller speed could be observed when the power input was kept constant (Figure 3B). The hydrolysis of arundo thus appears rather independent of both mixing power and impeller speed, a finding that corresponds well with previous results regarding different agricultural residues, e.g. corn stover [15] and wheat straw [16]. Since power input appears to have no significant impact on the hydrolysis of arundo and other similar materials (e.g. corn stover [15] and wheat straw [16]) a horizontal reactor, where power inputs can be kept rather low due to low rotation speeds, is likely well-suited for enzymatic hydrolysis of these types of material [9,16].

The influence of power input on spruce hydrolysis at different WIS contents was, however, shown to be significant. Performing the hydrolysis at the same power input at different WIS content resulted in a qualitative “shift” in the glucose yields from an increase in yield at higher WIS content (at constant impeller speed - Figure 1D) to a decrease in yield when increasing the WIS (Figure 4D). To be able to keep the same power input regardless of the WIS content a higher stirrer speed was needed at low WIS content (Figure 3D). By increasing the power input (and hence stirrer speed) for the lower WIS contents we were able to increase the yields substantially for the spruce at 10 and 15% WIS content (Table 3). This is in line with previous results were we found a strong influence of power input on spruce hydrolysis at 10% WIS [12] and additionally emphasizes the need to consider power input when designing and comparing hydrolysis experiments for spruce.

The difference in response to power input between spruce and arundo (and other materials) is not easily understood. A possible explanation could be that it is not the power input and stirrer speed per se, but rather the presence of high shear forces in the reactor that plays an important role. The shear forces in the reactor is the product between the viscosity and the shear rate, the latter is proportional to the stirrer speed [22]. The viscosity is generally higher for the spruce material throughout the hydrolysis, as seen in the torque profiles (Figures 2 and 3). The continued increase in stirrer speed during the spruce hydrolysis (Figure 3D) furthermore indicates that the enzyme degradation affects the viscosity of the material during a longer time compared to the arundo experiments where impeller speeds leveled off after a shorter time (Figure 3B). At elevated viscosities the shear forces might be strong enough to “peel-off” fiber fractions and hence create smaller fiber particles. This might explain the increased yield at high WIS content for spruce since the viscosity was much higher than at lower WIS contents. In contrast, the lack of a power input effect for the arundo may be connected to the rapid initial drop in viscosities down to levels that were almost WIS independent. This would be in line with the report of Samaniuk *et al.* of strong synergetic effects between mixing and enzymatic actions in terms of reduced particle sizes. They studied enzymatic hydrolysis of corn stover in high shear mixing systems (i.e. screw extruders) [23] and their experiments continued for 40 minutes and the reduction in particle size seemed to level off before that, possibly due to a too low viscosity. In addition, the higher shear forces could enhance cellulose degradation due to creation of more amorphous regions according to a hypothesis put forward by Lenting and Warmosken [24]. The creation of smaller particles or more amorphous regions on the cellulose might also enhance enzyme adsorption onto the material and hence increase the hydrolysis rate. Better enzyme adsorption at increased shear forces have previously been reported and attributed to mixing [25-27].

## Conclusions

In this study we investigated high solid hydrolysis of arundo and spruce in stirred tanks. By torque and power consumption measurements, large rheological differences between the materials were found. Most striking was the fact that mixing energy input was rather independent of initial WIS content for arundo, while the energy input was heavily influenced by the WIS content for spruce. Moreover, spruce glucose yields were shown to be strongly influenced by the mixing power whereas arundo was rather unaffected by changes in power input. Based on these findings we reasonably assume that hydrolysis equipment design and mode of operation likely will have to be feedstock specific, at least when considering such different materials as softwood (e.g. spruce) and agricultural residues/energy crops (e.g. arundo).

## Competing interests

The authors declare that they have no competing interests.

## Authors' contributions

BP participated in the design of the study, performed the experimental work, and wrote the manuscript. GL participated in the design of the study and commented on the manuscript. Both authors contributed to the scientific discussion throughout the work, and have read and approved the final manuscript.
